# High abundance of CD271^+^ multipotential stromal cells (MSCs) in intramedullary cavities of long bones

**DOI:** 10.1016/j.bone.2011.07.016

**Published:** 2012-02

**Authors:** George Cox, Sally A. Boxall, Peter V. Giannoudis, Conor T. Buckley, Tarek Roshdy, Sarah M. Churchman, Dennis McGonagle, Elena Jones

**Affiliations:** a Academic Department of Trauma & Orthopaedics, School of Medicine, University of Leeds, UK; b Academic Unit of the Musculoskeletal Diseases, Leeds NIHR Biomedical Research Unit, University of Leeds, UK; c Trinity Centre for Bioengineering, School of Engineering, Trinity College Dublin, Ireland

**Keywords:** Multipotential stromal cells, Mesenchymal stem cells, Long bone, Bone marrow, Intramedullary cavity, Bone regeneration

## Abstract

Aspiration of iliac crest bone marrow (ICBM) remains the most frequent technique used in harvesting multipotential stromal cells (MSCs) for bone regeneration. Although this tissue type is easily accessed by a surgeon, it has a low frequency of MSCs, which is significant given the high cell numbers required for bone regeneration strategies. Lipoaspirates possess higher MSC frequencies, albeit cells with a differentiation profile less suited to orthopaedic interventions. Intra-medullary cavities of long bones have previously been shown to harbour MSCs in animals, however evaluation of their frequency, differentiation capacity and phenotype in humans had not previously been performed.

Long bone fatty bone marrow (LBFBM) was collected prior to harvesting bone graft. Basic cellular compositions of donor-matched LBFBM and ICBM aspirates, including the numbers of CD34^+^ hematopoietic stem cells and CD31^+^ endothelial cells, were similar. MSCs were enumerated using colony-forming-unit-fibroblast assays and flow cytometry for the presence of a resident LBFBM CD45^−/low^ CD271^+^ MSC population and revealed a trend for higher MSC numbers (average 5 fold, n = 6) per millilitre of LBFBM compared to donor-matched ICBM. Functional characteristics of resident MSCs, including their growth rates, differentiation potentials and surface phenotypes (CD73^+^CD105^+^CD90^+^) before and after culture-amplification, were similar. Enhanced numbers of MSCs could be recovered following brief enzymatic treatment of solid fragments of LBFBM.

Our findings therefore reveal that the intramedullary cavity of the human femur is a depot of MSCs, which, although closely associated with fat, have a differentiation profile equivalent to ICBM. This anatomical site is frequently accessed by the orthopaedic/trauma surgeon and aspiration of the intramedullary cavity represents a ‘low-tech’ method of harvesting potentially large numbers of MSCs for regenerative therapies and research.

**This article is part of a Special Issue entitled: *Interactions Between Bone, Adipose Tissue and Metabolism*.**

## Introduction

The iliac crest bone marrow aspirate (ICBMA) was the first source from which multipotential stromal cells (MSCs), also termed mesenchymal stem cells, were isolated [1]. This anatomical site has become the most frequently accessed in harvesting MSCs for bone tissue engineering and is generally accepted as the ‘gold-standard’. Whilst this source is readily accessible and has good handling properties it has a low frequency of MSCs (0.001–0.01%) [1]. This is of significance as many regenerative medicine uses of MSCs including putative bone repair applications require large cell numbers [2–4]. High MSC yields can be achieved by *in vitro* culture with relative ease, with a 1000-fold increase in numbers within 2–3 weeks [5]. However, this results in daughter cells that have reduced differentiation capacity [5] and impaired cell function including gradual accumulation of senescence-related markers [6,7] and increased potential for transformation [8]. This process is also time-inefficient, expensive and requires industrial-scale good-manufacturing practice (GMP)-compatible cell expansion facilities and a two-stage surgery for tissue procurement and implantation [9].

Due to these factors there is a need to find alternative MSC sources where there is a potentially larger yield of cells. Initial techniques involved the development of devices to concentrate MSCs from large volumes of ICBM aspirate by centrifugation [10]— and such devices are available in the clinic. The implantation of 50,000 uncultured MSCs/CFU-Fs by concentrating up to 300 ml of ICBMA has shown an improvement of fracture healing in one study [10]. However, it is not always possible to obtain such large amounts of ICBMA. The enzymatic digestion of adipose-rich connective tissues such as fat has been proposed as an alternative strategy, with authors reporting the liberation of 500-fold more MSCs per gram of tissue when compared with ICBMA [11]. Lipoaspiration however cannot be performed for every orthopedic patient and the “quality” of lipoaspirate-derived MSCs for bone repair applications remains debatable [12–17].

Multipotential stromal cells have previously been shown to be present in the intramedullary cavity of long bones in humans [18]. However, this has been largely ignored as a source of MSCs for bone regeneration. In contrast, the harvesting of long-bone BM has been practiced on rat [19], mouse [20], rabbit [21–23] and pig [24,25] and is probably the most prevalent research method of isolating MSCs from animals. Analogous to other adipose-rich tissues, it may be hypothesized that the intra-medullary (IM) contents of long bones contain large numbers of MSCs. Unlike peripheral fat tissues, the MSCs are present in a bone related micro-environment and may potentially exhibit good intrinsic osteogenic capabilities. This is supported by early pioneering findings documenting a strong *in vivo* osteogenic capacity of adipogenic marrow cells [23].

This study explored the aspirated contents from the IM cavities of long bones, which are frequently accessed by the trauma/orthopaedic surgeon, as a source of MSCs in comparison to the ‘gold standard’ iliac crest aspirated source. We used colony-forming fibroblast (CFU-F) assay [26] and flow cytometry for CD45^−/low^CD271^+^ fraction [27–29] to enumerate MSCs and compared their frequency with donor-matched ICBM aspirates. We also used functional *in vitro* assays for MSC expansion and differentiation, to demonstrate that MSCs from IM cavities of long bones were equal or superior to their ICBMA counterparts. These findings should permit the development of novel one-step MSC harvesting procedures for bone repair augmentation in fracture patients.

## Materials and methods

### Subjects and specimen collection

Approval for these studies was obtained from the Leeds Teaching Hospital NHS trust ethics committee, with all patients providing informed consent. For the purpose of this research, iliac crest bone marrow aspirate (ICBMA) was obtained from 11 acute trauma patients, 11 patients with post-traumatic disorders, 15 patients with long bone (tibia/femur) non-union fractures and 6 healthy controls (Table 1). Samples were harvested from patients during operation; ICBMA (10 ml) was aspirated from the anterior iliac crest, using an 11-gauge, bevel-tipped trocar (Stryker, Kalamazoo, Michigan, US) and 10 ml syringe (BD Biosciences, Oxford, UK). Donor-matched ICBMA and LBFBM material was collected from 8 patients with non-union fractures (median age 35 years, range 19–65).

The LBFBM contents were aspirated via the greater trochanter, which was opened surgically, prior to the harvest of bone graft using the reamer–irrigator–aspirator (RIA) (Synthes, Westchester, Pennsylvania, USA) for the grafting of non-unions [30]. Following the operative opening of this cavity, via the greater trochanter, suction tubing and 50 ml bladder syringe was used to collect the sample. In some patients with fracture non-union 10 ml of peripheral blood (PB) was also collected (n = 5). Samples were transferred immediately into EDTA containing vacutainers (BD Biosciences) and transported to the laboratory.

### Specimen processing

Samples were processed under aseptic conditions and sample volumes in millilitres were recorded. The average sample volume for long bone fatty bone marrow aspirate was 12 ml (range 11–17 ml); ICBM aspirate volume was always 10 ml. A manual nucleated cell (NC) count was performed on every sample following red cell lysis in 4% acetic acid (Sigma, Gillingham, UK). In some experiments (n = 4), the aspirated IM contents were left at room temperature for 1 h, during which the fatty contents congealed leading to the formation of ‘solid’ and ‘liquid’ phases. To physically separate these phases, samples were passed through a 70 μm cell strainer (BD Biosciences). The resulting solid phase was digested using collagenase (Stem Cell Technologies, Grenoble, France) at 1:1 ratio w/v (final concentration = 0.125%), for 60 min at 37 °C. Subsequently all fractions were used for CFU-F assay or initiation of *in vitro* MSC cultures. In some experiments (n = 7), mononuclear cells (MNCs) were isolated using Lymphoprep (Axis-shield, Huntingdon, UK) and counted, as described previously [27].

### CFU-F assay and MSC expansion

CFU-F assay was performed as previously described [26] with minor modifications. Briefly, 100 μl or 200 μl of each sample (200 μl or 400 μl of the matched FBM-solid fraction to account for dilution with collagenase) was directly plated into a 10 cm diameter petri-dish (Corning Life Sciences, Amsterdam, Holland) with 15 ml of non-haematopoietic media (Miltenyi Biotec, Bisley, UK) in duplicate. Cells were allowed 48 h to adhere, after which red blood cells and other non-adherent cells were removed with two washes of phosphate buffered saline (PBS) (Invitrogen, Paisley, UK). Adherent cells were cultured (37 °C, 5% CO_2_) with half-media changes performed twice weekly. PB MNCs were seeded at 5 × 10^6^ cell/dish and cultured similarly [31]. Crystal violet staining was performed on the fourteenth day, as previously described [32] before scoring blindly.

Expansion was performed to produce sufficient cells to undertake trilineage differentiation and cell surface phenotyping in all fractions as previously described [32]. Cells were expanded until 80% confluency was attained (denoted as passage 0/P0), after which cells were trypsinised and passaged up to P3 [32,33]. Population doublings (PDs) were calculated according to the following formula: PDs = log^2^(N total cells / Total CFU-F on day 0) [33].

### Tri-lineage differentiation

Passage-3 MSCs (n = 4 donors) were induced towards osteogenesis, chondrogenesis and adipogenesis according to standard protocols [1,32]. For osteogenesis, cells were seeded at a density of 3 × 10^4^/well in 3 cm diameter wells (Corning Life Sciences) and cultured in low glucose DMEM with 10% FCS, supplemented with standard antibiotic mixture (100 U/ml penicillin and 100 μg/ml streptomycin) (all from Invitrogen), 100 nM dexamethasone, 10 mM β-glycerophosphate and 0.05 mM ascorbic acid (all from Sigma), with twice weekly half-media changes. Alkaline phosphotase activity was assessed on day 14 post-induction, as previously described [32]. For adipogenesis, cells were seeded in 12-well plates at 1 × 10^5^ cells/well and cultured in low glucose DMEM with 10% FCS, antibiotics, 10% horse serum (Stem Cell Technologies), 0.5 mM isobutylmethylxanthine, 60 μM indomethacin and 0.5 μM hydrocortisone (all from Sigma). Cultures were stained on day 14 post-induction with Oil-Red-O, as previously described [27,32].

A 3D pellet culture model was used to induce chondrogenesis as previously described [32] with minor modifications. Briefly, pellets were formed in 1.5 ml micro-centrifuge tubes by centrifugation (650 *g*, 5 min) of 2.5 × 10^5^ cells suspended in 1 ml of serum-free medium consisting of high glucose DMEM (Invitrogen), antibiotics, 40 μg/ml l-proline, 1.5 mg/ml BSA, 4.7 μg/ml linoleic acid, 1× insulin–transferrin–selenium, 50 μg/ml l-ascorbic acid-2-phosphate, 100 nM dexamethasone (all from Sigma) and 10 ng/ml TGF-β3 (R&D Systems, Abbingdon, UK). Full media changes were performed twice weekly and biochemical assessment performed at 21 days as previously described [34] with minor modifications. Briefly, pellets were digested for 18 h at 60 °C, with a papain digestion solution containing 100 mM Sodium Phosphate Buffer supplemented with 5 mM Na_2_EDTA, 10 mM l-cysteine and 0.125 mg/ml papain (all from Sigma). DNA content was assessed using a Quant-iT™ PicoGreen® dsDNA Reagent Kit (Invitrogen) and produced glycosaminoglycan (GAG) was measured using a Blyscan™ kit (Biocolor Life Sciences, Co Antrim, Ireland).

### Surface phenotype of culture expanded MSCs

Passage-3 MSCs (n = 3 donors) were trypsinised and re-suspended at 10^7^ cells per ml in FACS buffer (PBS + 0.5% BSA) before surface staining with CD45 (Leukocyte Common Anigen)-PE-Cy7, CD34 (gp105–120)-PerCp (BD Biosciences), CD271 (Nerve growth factor receptor)-APC (Miltenyi Biotec), and a pair of FITC- and PE-labelled antibodies, including: CD31(PECAM-1)-FITC (Serotec, Kidlington, UK), CD33 (gp67)-FITC, CD61 (Integrin β3)-FITC, CD73 (5′ Ecto-nucleotidase)-PE , CD19-PE (BD Biosciences), CD105 (Endoglin)-PE and CD90 (Thy1)-PE (Serotec). All antibodies were used at the manufacturers' recommended concentrations with matched isotype controls (from Serotec). Dead/dying cells were excluded from the analysis using DAPI (Sigma) and were normally less than 5%. Data were analysed on an LSRII flow cytometer equipped with DIVA software (BD Biosciences).

### MSC enumeration

The phenotypic identification of the ‘*ex-vivo* MSC’ using the CD45^−/low^CD271^+^ phenotype was first described by our group using ICBMA [27,28,35] and has since been independently validated by others [29,36,37]. MSC enumeration was performed by staining the aspirated MNC fraction with CD45-FITC (Dako UK Ltd, Ely, UK), CD271-PE (Miltenyi Biotec) and 7-AAD, as previously described [28]. A minimum of 5 × 10^5^ events were acquired and analysed using an LSRII flow cytometer to establish the percentage of CD45^−/low^CD271^+^ cells. The frequency of CD45^−/low^CD271^+^ per ml of sample was then calculated based on the following formula: CD45^−/low^CD271^+^ cells/ml = % CD45^−/low^CD271^+^ cells × MNCs/ml.

### Extended phenotypic analysis of ‘*ex-vivo* MSC’ using 6-colour flow cytometry

Bone marrow MNCs were isolated using Lymphoprep and cells were then re-suspended at 1 × 10^7^ cells/ml in FACs buffer. Antibodies were added at the manufacturers' recommended concentrations and the cells were incubated for 20 min. Antibodies used were: CD45-PECy7, CD73-PE, CD34-PerCP, CD19-PE, CD33-FITC, CD61-FITC (BD Biosciences), CD90-PE, CD105-PE, CD31-FITC (Serotec) and CD271-APC (Miltenyi Biotec). The cells were washed and re-suspended in FACs buffer containing 100 ng/ml DAPI before analysing on an LSRII flow cytometer. Dead cells were excluded from the analysis using DAPI (usually < 5%) before gating on the CD45^−/low^ CD271^+^ cell population and assessing the expression of all other markers.

### Statistics

Statistical analysis and graphing were performed using GraphPad Prism version 4 for Windows (San Diego, California, USA). Gaussian distribution could not be assumed given the number of samples and differences between donor-matched ICBMA and LBFBM groups were tested using Wilcoxon signed ranks test. The differences in the MSC content between different patient groups were analysed using Mann–Whitney test. Significance was assumed when p < 0.05.

## Results

### Basic cellular characterisation of ICBM and LBFBM aspirates

A standard CFU-F assay was first performed to measure the MSC content of ICBM aspirates in three groups of orthopaedic patients and healthy controls (Table 1). Consistent with previously reported findings [10], high donor-to-donor variation was observed, potentially due to factors related to donor age [38] or a variable degree of dilution of ICBM sample with blood during the aspiration procedure [39]. No significant differences in CFU-F abundance in ICBMA were found between all three groups of orthopaedic patients and healthy controls (Table 1). A flow cytometry-based ‘instant’ enumeration of MSCs was additionally performed based on their CD45^−/low^CD271^+^ phenotype. Consistent with our previous data [28], direct positive correlation was observed between the numbers of CFU-Fs and CD45^−/low^CD271^+^ cells per ml of ICBMA (r = 0.700, p = 0.013, n = 13). These data confirmed a possibility of using flow cytometry for enumerating MSCs in other marrow sources, including LBFBM aspirates.

**Fig. 1:**
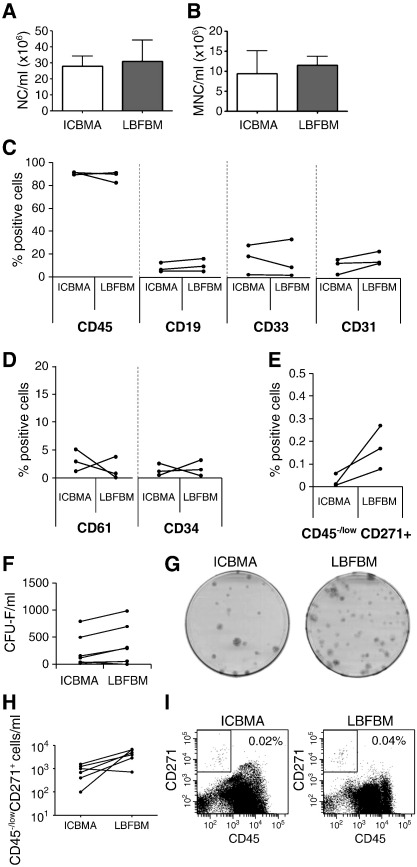
Cellular characterisation of ICBM and LBFBM aspirates. A — Total nucleated cell count in ICBMA (white bar) and LBFBM (grey bar, n = 9) after red cell lysis. B — Mononuclear cell count in ICBMA (white bar) and LBFBM (grey bar, n = 7) after MNC isolation using a density gradient. C — Proportions of leukocytes (CD45), B cells (CD19), myeloid cells (CD33) and endothelial cells (CD31) in MNC fraction. D — Proportions of megakaryocytic (CD61) and hematopoietic progenitor (CD34) cells. E — Proportions of CD45^−/low^CD271^+^ MSCs. F — Total numbers of CFU-Fs per ml. G — CFU-F dishes from a representative donor showing increased numbers of colonies in LBFBM. I — Total numbers of CD45^−/low^CD271^+^ cells per ml. H — Flow cytometry plots from a representative donor showing increased numbers of CD45^−/low^CD271^+^ cells in LBFBM. Donor-matched samples are used in all experiments, error bars represent SDs.

The analysis of different hematopoietic and non-hematopoietic cell types in ICBM and LBFBM aspirates was first performed to compare their basic cellular composition (Fig. 1). The cellularity (both as total NC and MNC counts) of LBFBM aspirates was similar to donor-matched ICBM aspirates (Figs. 1A and B). The majority of cells in both tissues were CD45^+^ leukocytes, including CD19^+^ B-cells, CD33^+^ myeloid cells and CD61^+^ megakariocytes/platelets (Figs. 1C and D). Similar to other cell types, the numbers of cells with pro-healing capabilities: CD34+ hematopoietic progenitor cells and CD31+ endothelial/angiogenic cells [40] were not statistically different between the two sources (Figs. 1C and D).

Resident MSCs were measured using CFU-F assay and flow cytometry for the CD45^−/low^CD271^+^ cell population (Figs. 1E–I). The frequency of CD45^low^ CD271^+^ cells was higher in LBFBM aspirate (Fig. 1E). In correspondence, LBFBM aspirate contained higher numbers of CFU-Fs compared to ICBMA (median values 293 and 115 CFU-F/ml, respectively), however differences narrowly failed to reach statistical significance (p = 0.0515, Fig. 1F). CFU-F dishes from a representative donor are shown on Fig. 1G. A similar trend for the MSC increase in LBFBMA was observed following the measurements of CD45^−/low^CD271^+^ cells/ml (Fig. 1H). Flow cytometry data from a representative donor are shown in Fig. 1I. It is noteworthy, that no CFU-Fs/MSCs were found in PB of patients with fracture non-unions (n = 5). Based on these findings it is evident that LBFBM aspirates were not inferior to ICBMA in terms of the proportions of regenerative cells and MSCs per sample volume.

### Extended phenotypic characterisation of uncultured MSCs in LBFBM aspirates

**Fig. 2:**
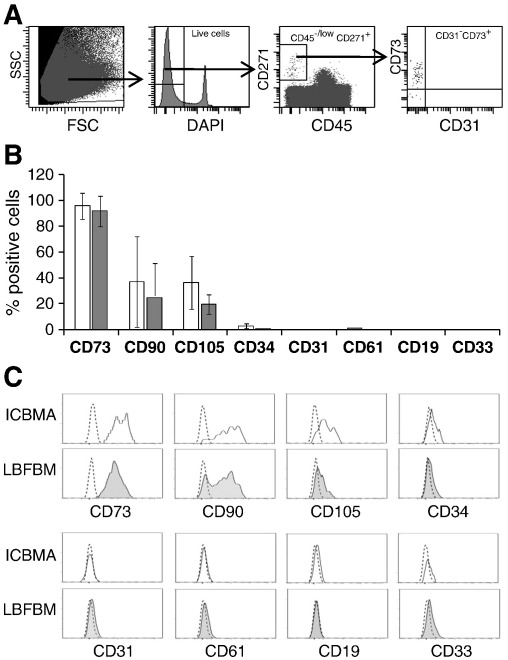
Phenotypic characterisation of ‘ex vivo’ MSCs in ICBM and LBFBM aspirates. A — Gating strategy: MNCs were gated on FSC/SSC to exclude cell debris and DAPI exclusion was used to remove non-specific fluorescence by dying/dead cells. The CD45^−/low^ CD271^+^ cells were then gated as the population of interest and expression of other markers (CD73 and CD31 shown as examples) was characterised within this population. B — The expression of classic MSC markers CD73, CD90 and CD105 and the lack of CD34, CD31, CD61, CD19 and CD33 on gated CD45^−/low^CD271^+^ cells from ICBMA (white) and LBFBM (grey)(n = 3 matched donors, error bars represent SDs). C — Histograms showing the expression of markers on gated CD45^−/low^CD271^+^ cells from ICBMA (white) and LBFBM (grey) from one representative donor. Dotted lines show isotype control.

Although MSCs were found in similar proportions in LBFBM and ICBM aspirates, their functional and phenotypic characteristics could be altered in fatty environments. An extended phenotypic analysis of CD45^−/low^CD271^+^ ‘ex vivo’ MSCs in LBFBM and ICBM aspirates was undertaken to identify any potential differences in surface receptor expression. The gating strategy for this analysis is shown in Fig. 2A. CD73 (5′ Ecto-nucleotidase) is a broadly-accepted MSC marker [1,39] and it was expressed at similar levels on CD45^−/low^CD271^+^ ‘ex vivo’ MSCs from both sources (~ 91%, n = 3) (Fig. 2B). The MSC markers CD105 (Endolgin) and CD90 (Thy1) were expressed at similar levels in LBFBM and ICBM aspirates (Fig. 2B) whereas CD31 (PECAM-1), an endothelial cell marker, was negative. Finally, we investigated the expression of CD34 molecule on MSCs from ICBMA and LBFBM. This was based on recently-published evidence of CD34 being present on MSCs from lipoaspirates [41]. Consistent with our previous findings relating to ICBMA [27], CD34 was absent on CD45^−/low^ CD271^+^ MSCs from both bone sources (Fig. 2B). Overall, MSC marker expression levels were similar in LBFBM and ICBM aspirates and representative marker histograms are shown on Fig. 2C. Therefore, based on the expression of 5 selected surface markers, CD45^−/low^ CD271^+^ cells from LBFBM aspirates had classical ‘ex vivo’ BM MSC phenotype, similar to ICBMA and different from lipoaspirates.

### Phenotypic characterisation of MSC cultures generated from LBFBM and ICBM aspirates

**Fig. 3:**
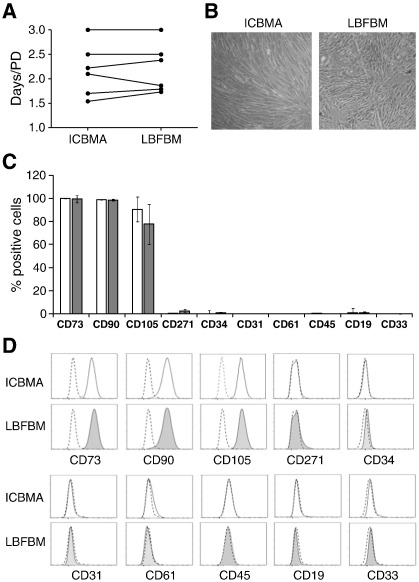
Phenotypic characterisation of MSC cultures generated from ICBM and LBFBM aspirates. A — MSC growth rates and B — morphological appearance (×40 magnification). C — Classic MSC phenotype of cultures generated from ICBMA (white) and LBFBM (grey) (n = 3 matched donors, error bars represent SDs). D — Histograms showing the expression of markers on MSCs cultured from ICBMA (white) and LBFBM (grey) from one representative donor. Dotted lines show isotype control.

Although MSC numbers and phenotypes were similar in ICBM and LBFBM aspirates, functional differences in MSCs could exist, due to their anatomical locations. We next compared growth and phenotypic characteristics of MSC cultures obtained from LBFBM and ICBM aspirates (Fig. 3).

No statistically significant differences were found in the growth rates, measured as days/PD up to P3, of ICBMA and LBFBM derived MSC cultures (median values of 2.36 and 2.44, respectively, Fig. 3A). Early-passage cultures (P3) from both sources had indistinguishable morphology (Fig. 3B) and similar phenotypes, using an extended panel of 10 surface markers (Fig. 3C). The majority of cultured cells expressed MSC markers CD73, CD90 and CD105 and were negative for hematopoietic lineage cell markers as well as CD31 and CD34. Representative histograms are shown on Fig. 3D. Altogether these data showed that LBFBM aspirates were similar to donor-matched ICBM aspirates in terms of growth and phenotypic characteristics of resident MSCs.

### Differentiation potentials of MSCs derived from LBFBM and ICBM aspirates

**Fig. 4:**
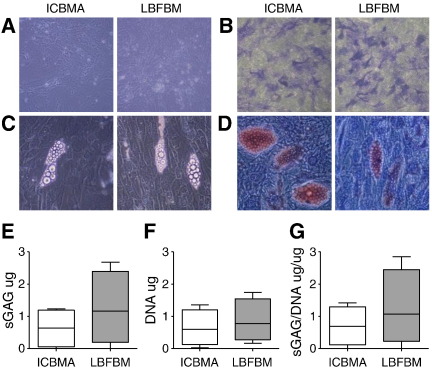
Differentiation potentials of MSCs derived from LBFBM aspirates. Day 14 osteogenesis: A — Unstained and B — stained for alkaline phosphatase (microphotographs ×40 magnification). Day 21-adipogenesis: C — Unstained and D — stained for Oil Red (microphotographs ×100 magnification). Day 21 — chondrogenesis: E — Amount of glycosaminoglycan (GAG) produced by the digested pellet cultures. F — Amount of DNA present in each pellet culture. G — To account for the varying cell growth within the pellet cultures the amount of glycosaminoglycan (GAG) produced per μg of DNA in the pellet was calculated.

To investigate tripotentiality, P3-MSC cultures derived from ICBM and LBFBM aspirates were placed in osteo-, adipo- and chondrogenic differentiation conditions (n = 4 donors)(Fig. 4). All cultures exposed to osteogenic induction conditions for 14 days contained polygonal cells consistent with osteoblastic progression (Fig. 4A). No obvious pattern of differences between ICBM and LBFBM aspirates was documented in the proportions of alkaline-phosphatase positive cells (Fig. 4B). Similar data were obtained for adipogenesis: all MSCs were able to produce Oil-Red positive mature adipocytes, with no apparent gross differences between the samples (Figs. 4C and D). Chondrogenesis was performed using a classical pellet culture [27] and measured as accumulation of cartilage-specific proteoglycans per cell [34]. Similarly to osteo- and adipogenesis, no significant differences between ICBM- and LBFBM-derived pellets were found (Figs. 4E–G).

We next investigated whether any observed donor-to-donor differences could be attributed to the “in vitro age” of tested cultures (measured as total PDs at P3, i.e. prior to differentiation). On average, MSCs from ICBM aspirates and LBFBM aspirates have both undergone 16PDs, with no apparent correlations being found between the “in vitro age” and functional outcomes for individual cultures. Overall, all these functional data showed that LBFBM MSCs were osteogenic and chondrogenic at the levels comparable, and often superior, to ICBM MSCs, making them suitable for therapeutic bone repair applications.

### Enhanced release of MSCs from LBFBM aspirates using an enzymatic digestion technique

**Fig. 5:**
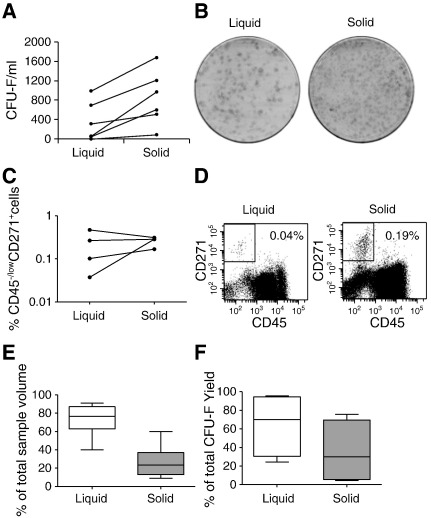
Enzymatic release of MSCs from LBFBM aspirates. A — Total numbers of CFU-Fs per ml. B — CFU-F dishes from a representative donor showing increased numbers of colonies in the solid fraction. C — Percentages of CD45^−/low^CD271^+^ cells in MNC fraction. D — Flow cytometry plots from a representative donor showing increased numbers of CD45^−/low^CD271^+^ cells in the solid fraction. Relative volumes (E) and relative CFU-F yields (F) in liquid and solid fractions (n = 4 donors, error bars represent SDs).

Following 1-hour storage at RT, fatty components of LBFBM aspirates tend to congeal, resulting in the formation of fatty solid aggregates. To extract increased numbers of MSCs from this material, the solid aggregates from LBFBM aspirates were exposed to a brief enzymatic digestion (Fig. 5).

Although a trend for higher numbers of CFU-F/ml was found in the solid phase (Figs. 5A and B), the differences were not statistically different between liquid and solid phases. Similar findings were observed for percentages of CD45^−/low^CD271^+^ cells (Figs. 5C and D). Fatty solid aggregates contributed to ~ 23% of total sample volume (Fig. 5E) and contained the equivalent of ~ 30% of the total sample's CFU-Fs (Fig. 5F). At room temperature these MSCs are “trapped” in the solid fatty aggregate, but were easily released by a brief enzymatic digestion. Alternatively, samples could be kept at body temperature (or at 37 °C in the laboratory) to avoid the loss of MSCs due to solidification of fatty components.

## Discussion

The conversion of red marrow to yellow marrow is a physiologically dynamic process that starts in infancy at the terminal phalanges and progresses in a centripetal direction [42], so that by adulthood the diaphyses of long-bones are almost entirely populated by yellow, fatty bone marrow [43]. MSCs are commonly harvested from long-bones in rat [19], mouse [20], rabbit [21,23] and porcine [24,25] models. In contrast to human subjects, the description of a yellow fatty appearance of the long bone marrow in these reports is rarely mentioned, which may be partly due to the fact that the majority of animal models are sacrificed at a juvenile stage — possibly prior to red marrow conversion. The aim of this study was to comprehensively assess human LBFBM as a source of MSCs for bone repair applications and to compare it with ICBM aspirate.

Using donor-matched samples, we have found that LBFBM was non-inferior to ICBMA in terms of its cellularity, basic cellular composition and the proportions of MSCs. In fact, LBFBM had higher proportions of CFU-Fs compared to ICBMA (2.5-fold). These differences narrowly failed to reach statistical significance but in a larger scale study they may do so. Despite the fatty environment within LBFBM cavity, LBFBM-derived MSCs possessed the classical MSC phenotype, before and after culture, arguing for good preservation of their undifferentiated status. Furthermore, LBFBM-derived MSCs had similar growth characteristics and multipotential properties as their ICBMA counterparts. This is of interest as MSCs from other adipogenic sources have often been shown to be inferior to ICBMA in forming bone [12,13] and this may be related to the intra-osseous location of MSCs in long-bone cavities.

Numerous literature reports describing the presence of CFU-F/MSCs in connective adipose tissues, including lipoaspirates, document MSC frequencies that are considerably higher than in BM aspirates (at least 2 orders of magnitude) (reviewed in [44]). In comparison, the abundance of MSCs in “yellow” fatty marrow aspirates observed in our study appears to be relatively minor (only a 2–5 fold higher than in classical “red” marrow aspirates). Given the unique function of adipocytes in the marrow [45–47] and the different metabolic functions of fat in different depot sites [47], our data indicate that the MSC pool size in “fatty tissues” is clearly site-specific. Variations in MSC function have been documented for different types of bone: orofacial, axial and appendicular [48] and different depots of fat: arm, flank, thigh and abdomen [49]. The heterogeneity of MSCs resident within seemingly the same type of tissues but located in different anatomical areas may be explained by varying local demands for tissue turnover and mechanical loads [48]. Additionally, the MSC topography in diverse human tissues has been described as primarily perivascular [50,51] and it is possible that the lower MSC frequency in fatty marrow as opposed to subcutaneous fat may be also related to blood vessel density as suggested previously for human synovium [52] and equine adipose tissue [53].

The fact that LBFBM-derived cultured MSCs were able to effectively differentiate towards osteoblasts and chondrocytes *in vitro* provided strong evidence that minimally expanded LBFBM-derived MSCs can be used as cell therapy for fracture non-unions. Furthermore, high numbers of CD45^−/low^CD271^+^ cells present in LBFBM samples (up to 67,000, median 43,620 in 10 ml) suggested that their direct injection, in a one-stage procedure, may be possible without prior cell-culture. One previous study has showed that a dose of 50,000 uncultured MSCs from ~ 300 ml of ICBMA was efficacious following injection into non-union fracture sites [10]. A lower volume of LBFBM would therefore be sufficient to obtain a similar number of MSCs. Uncultured MSCs could be effectively concentrated using magnetic beads against the CD271 molecule, based on our findings showing that the proportions of CD45^−/low^ CD271^+^ cells closely reflected that of CFU-Fs [28].

The findings from this study also offer an additional cellular mechanism to explain the efficient bone healing process following LB fracture. They show, for the first time, that the marrow contents of long bones contain large numbers of functionally-competent local MSCs. Given a novel concept of local MSC recruitment to fracture sites [54,55] and our findings showing large numbers of MSCs in LBFBM in humans, our data point towards a potentially major contribution of locally-recruited LBFBM MSCs to healing of long bone fractures. Systemic MSC circulation in healthy humans and in response to injury remains poorly understood [56–60], and in this respect our findings showing no circulating MSCs in patients with fracture non-union (despite high MSC numbers in ICBM and LBFBM) are noteworthy. To the best of our knowledge, the enumeration and the ‘ex vivo’ phenotype of MSCs in LBFBM in comparison to donor-matched ICBMA have never been reported before. Furthermore, we showed that the marrow contents of long bones contained normal amounts of other cells with regenerative potential (CD34^+^ and CD31^+^ cells [61,62]) necessary to orchestrate the fracture healing processes.

In summary, this study demonstrates that the femoral IM cavity represents a depot of MSCs which could be used for autogenous/allogeneic use and can be harvested using ‘low-tech’ techniques for a variety of commonly performed operations including trauma surgery and total hip replacement. The IM cavities of long-bones, in which the FBM resides, are also readily accessible by the orthopaedic surgeon during lower-limb arthroplasty/nailing of long-bone fractures, with the marrow contents requiring removal prior to prosthesis insertion. Enumeration of MSCs from LBFBM is possible using the CD271^+^ CD45^low^ phenotype and their concentration could be achieved with the use of magnetic beads against the CD271 molecule. The use of freshly-isolated or minimally-expanded LBFBM-derived MSCs could therefore have important scientific and economic benefits in tissue engineering and treatment of fracture non-unions.

## Conflict of interest

The authors declare that there is no conflict of interest.

## Figures and Tables

**Table 1:** Characteristics of patients participating in this study.

Patient group	Diagnosis	Procedure	Time of procedure after initial injury, median (range)	Age, years, median (range)	CFU-F/ml of ICBMA, median (range)
Acute trauma (n = 11)	Fracture of pelvis (n = 10) Fracture of tibial plateau (n = 1)	Open reduction and internal fixation (n = 11)	3 days (0–10 days)	42 (21–72)	48 (11–1500)
Post-traumatic disorders (n = 11)	Post traumatic sacroiliac joint instability (n = 2) Other (n = 9)^a^	Injection (n = 2) Removal of metal (n = 2) Other^b^ (n = 7)	3 months (1–48 months)	33 (18–67)	90 (6–1445)
Established atrophic non-union (n = 15)	Fracture non-union (femur, n = 9, tibia, n = 6)	Reamer-Irrigator aspiration	18 months (6–72 months)	39 (19–65)	139 (8–2110)
Normal controls (n = 6)	NA	ICBM harvest for allogeneic transplant	NA	35.5 (19–58)	148 (48–1943)

NA — not applicable.

a Include post traumatic heterotrophic bone formation, infection, meniscal injury, and coccydynia.

b Include excision, debridement, and arthroscopy.
